# Editorial: Health is a Political Choice

**DOI:** 10.1093/eurpub/ckae044

**Published:** 2024-04-03

**Authors:** Charlotte Marchandise, Hans Henri P Kluge, Ricardo Mexia, Floris Barnhoorn

**Affiliations:** EUPHA Executive Director; EUPHA Executive Director; Chair of the 17th EPH Conference, Lisbon 2024; Deputy Director EPH Conference

Dear readers,

The European Public Health Association (EUPHA) is committed to advocating for evidence-based policy. However, despite longstanding dialogue about prioritizing health on political agendas, progress remains insufficient. This is sadly highlighted by the European Council's budget reductions in health, announced on 1 February 2024. 

The question then arises: How do we transform the decision-making process? Together with our partners—WHO Regional Office for Europe, European Commission and Austrian Public Health Association—we have chosen ‘Health is a Political Choice’ as the overarching theme for the European Public Health Week from 13 to 17 May.

We will open the dialogue online and in person in Brussels on Monday, at the European Observatory on Health Systems and Policies, discussing key subthemes that are critical to integrating health into the political agenda effectively. These include addressing health within the context of crises and exploring the concepts of a well-being economy and politics. Additionally, we wll delve into the importance of community participation in health politics, the implementation of Health in All Policies, and the broader application of health considerations across all policy areas to truly achieve Health for All Policies. This day aims to set the stage for a week of fruitful discussions, laying the groundwork for actionable insights and strategies to ensure health is prioritized across all levels of political decision-making.

A primary focus will be on equipping researchers and practitioners with the advocacy skills necessary for impactful community management, bold decision-making, and collaborative advocacy among Europe's health actors. Our goal is to amplify our collective voice, ensuring it resonates with decision-makers, the media, and citizens. Plans are underway to organize regular trainings to enhance these critical skills and competencies.

The Public Health Practitioners, working daily with the communities, constitutes our core allies. Although they may not regularly be able to publish articles or attend the EPH conference, their efforts across all health determinants are indispensable. We are committed to providing them with greater visibility, such as dedicating Tuesday to ‘Mental Health: People at the Center’, and highlighting citizen-based science and interventional research. The mobilization of Public Health Voices in Politics—encompassing patients, individuals with disabilities, members of discriminated communities, the LGBTQ+ community, and migrants—is paramount. EUPHA’s Sections are gathering research and organizing events, contributing to the broader dialogue on public health challenges and solutions, thereby enriching our collective understanding and driving forward the agenda for a healthier Europe. We are inviting them to publish more often on these columns.

While international and national political figures often capture our attention, the significance of local-level politics in public health cannot be understated. Wednesday's ‘Glocal’ approach—merging global insights with local actions—will feature contributions from WHO Healthy Cities and Regions for Health, emphasizing the power of localized initiatives.

The ‘Inspiring Doctor’ podcast by the British Medical Association, featuring Hannah Barham-Brown and invited by Martin McKee (https://www.bma.org.uk/inspiring-doctors-podcast), underscores the necessity and benefits of political involvement. The health and social crisis amplifies the need for informed voices within the political sphere. We all know figures who demonstrate the significant impact public health professionals can have on health policy—often at local level: let’s invite them, empower and support them to work closer together. 

Thursday’s theme, ‘Health through the Life Course: People at the Center, Breaking Down Silos from Childhood to Ageing’, underscores the imperative of political choices in shaping health outcomes across all stages of life. This day is dedicated to exploring the continuum of care necessary for a holistic health journey, emphasizing the need for universal health coverage and seamless access to medicines. Ensuring that every individual, regardless of age, orientation, or origin, has access to the care and resources they need to thrive require to break down the (political) barriers that impede our progress towards a more inclusive, equitable health system.

While we frequently discuss the importance of young voices, it's time to reflect on our tangible actions. Amplifying these voices means more than just acknowledging their value; it involves actively relinquishing control, sharing the stage, speaking less, and handing over the microphone. The final day of the European Public Health Week will focus on Youth Leadership, led by EUPHAnxt and co-organized with other youth associations. Our dedication lies in supporting those prepared to embrace the actual and upcoming challenges, a commitment echoed in Hans Kluge’s editorial.

Empowering advocacy, engaging politicians directly, and mobilizing all public health voices in the political sphere are key ensure that health is not only a choice but a priority for our leaders. We invite you to join us for the European Public Health Week, an event rooted in the principles that define EUPHA: a steadfast commitment to evidence-based information, a dedication to reducing health inequalities, a pursuit of collaborative efforts within and beyond the public health arena, and a relentless focus on promoting diversity and inclusion.

## Charting a path to a sustainable future, with young people

KlugeHans Henri P
EUPHA Executive Director

EJPH

The interlinkages between different environmental risks result in at least 1.4 million deaths per year in the WHO European Region. Extreme weather events, rising sea levels, depleting water resources and heat stress, pose a very significant direct threat to public health. They also amplify the effects of pollution, from air and water contamination to hazardous chemicals, upon people across the Region. At the same time, the loss of biodiversity, disrupting the intricate web of interdependent life that underpins ecosystems, undermines the very foundations of human health, driving the potential for future pandemics and aggravating the threat that antimicrobial resistance poses.

This speaks to the hearts and minds of young people: in a recent global study of more than 10 000 young people aged 16–25 years in 10 countries, nearly 60% reported being ‘very or extremely worried about climate change’. Moreover, nearly half admitted that these feelings impact their daily life and more than three-quarters felt betrayed by adults and governments by their apparent inaction (https://www.nature.com/articles/s44220-023-00059-3).

The fight for a liveable future hinges not just on the present but on the legacy we leave for our children and grandchildren. In this fight, the voices of young people are indispensable—because their reality is the climate and environmental crisis they inherit from us.

We owe it to young people, as a matter of intergenerational justice and for the sake of our vision for a healthy future, to empower them as agents of change. For this reason, WHO/Europe has established the Youth4Health network as a new initiative to amplify youth voices and perspectives into all areas of our work. By including them in decision-making, supporting their climate initiatives and echoing their concerns, we seek to ensure their voices are heard and their actions contribute to a sustainable, healthy future.

Their creativity, and passion can bridge the gap between knowledge and action, turning anxieties into solutions. Empowering young people and giving them representation and agency, however, is not enough. Mindful of the need to proactively recognize and address these legitimate concerns and expectations, European and Central Asian ministers gathered in July 2023 in Budapest to adopt the Declaration of the Seventh Ministerial Conference on Environment and Health, subsequently endorsed by the 73rd session of the WHO Regional Committee for Europe.

This landmark Declaration is a testament to the Region's strong commitment to good environmental stewardship for healthier populations. It outlines a comprehensive roadmap for addressing the triple crisis of climate change, environmental pollution, and biodiversity loss while fostering healthy, equitable, and sustainable communities.

Recognizing the interconnectedness of human, animal, and environmental health and integrating these considerations into its ‘Roadmap for healthier people, a thriving planet and a sustainable future’, the Declaration charts a transformative path towards addressing these challenges, including by supporting the operationalization of the One Health and Planetary Health approaches.

Young people were invited to the table in Budapest. We saw their determination with the establishment of a new Partnership on Youth whose aim is to provide a pathway for a more active role in shaping the future by participating in all environmental and health processes.

As we move forward in implementing what has been committed to, one overarching imperative will demand razor-sharp focus to ensure intergenerational equity and a liveable planet for the generations to come: climate action.

Inaction is not an option, and partnerships are a key ingredient of the action needed. At COP28 in Dubai we launched the Transatlantic Initiative on climate change and health: WHO/Europe and the Pan American Health Organization (PAHO) in partnership with the United States Department of Health and Human Services are establishing a joint programme of policy dialogues and a transatlantic conference to facilitate the exchange of knowledge and experience across the Atlantic on many of the common challenges on climate–health adaptation, mitigation, and preparedness, benefitting from the diversity of the climate change and health needs, experiences, and solutions.

Together with the Partnership for Health Sector Climate Action, established under the Budapest Declaration, we will in 2024 bring together health sector and other stakeholders to share best practices and mobilize joint action and resources so that we can support Member States of both regions to achieve the commitments taken through the COP28 Climate and Health Declaration—signed by 143 countries—and accelerate the transition towards more equitable climate-resilient communities and health systems.

Both processes are complementary and synergistic to the WHO-led Alliance for Transformative action on Climate and Health—a global mechanism positioned as the implementation vehicle to take forward the goals of the COP28 Climate and Health Declaration agenda with over 80 participating countries.

In May this year, 16 years after the first resolution, WHO Member States are expected to adopt a new World Health Assembly resolution on climate change and health; a resolution that is to seek to urgently address the magnitude of the climate crisis impact on health.

The path ahead of us is clear—if we are determined not to fail the generations to come. Let us build a future where hope flourishes alongside resilient communities, health systems and governance.

*Declaration of the Seventh Ministerial Conference on Environment and Health: Budapest, Hungary 5–7 July 2023*

## Sailing the waves of European public health: exploring a sea of innovation

MexiaRicardo
Chair of the 17th EPH Conference, Lisbon 2024
BarnhoornFloris
Deputy Director EPH Conference

The 17th EPH Conference, set to convene in Lisbon from 12 to 15 November 2024, is themed ‘Sailing the waves of European public health: exploring a sea of innovation’. This theme symbolizes our collective endeavour in the public health domain, mirroring the spirit of ancient explorers who ventured into unknown waters. Considering the European Commission has named Lisbon as this year's European Capital of Innovation, we will be at the perfect setting.

The conference will have five subthemes:

*Artificial Intelligence in Public Health:* Lisbon, where the past seamlessly intertwines with the future, provides an ideal setting to examine the groundbreaking role of Artificial Intelligence (AI) in public health. In this session, we will explore how AI is transforming disease surveillance, enhancing healthcare delivery, and refining decision-making processes, marking the dawn of a new era in health innovation. Coinciding with the Web Summit Lisbon 2024, we are actively exploring collaborative opportunities in this domain to further enrich our discussions and insights.

*One Health—Animal Approach:* Drawing inspiration from Lisbon’s rich maritime history, the conference will highlight the One Health concept, emphasizing the integral connection between human, animal, and environmental health. Discussions will focus on how an animal health perspective can enhance public health strategies and counteract emerging health threats.

*Social Marketing in Public Health:* Set against the lively streets and dynamic culture of Lisbon, this theme will explore the power of social marketing in public health. Participants will examine successful campaigns, digital strategies, and community engagement efforts aimed at fostering healthier lifestyles.

*Health Inequalities:* The varied neighbourhoods of Lisbon, each with its distinct identity and set of challenges, underscore the persistent issue of health inequalities across Europe. The conference will address innovative approaches to minimize these disparities and promote equal access to healthcare services.

*Global Health:* Reflecting on Lisbon’s role as a pivotal global port, this theme will discuss the significance of European public health initiatives in the broader context of global health. The aim is to tackle health issues that know no borders, emphasizing our shared responsibility in fostering a healthier global community.

The Conference boasts an extensive programme, featuring the traditional 16 tracks designed to broaden the scope of public health knowledge and foster numerous networking opportunities. Additionally, we are planning a diverse array of pre-Conference events, details of which are forthcoming. Among these, we are excited to announce the ‘*Conferência de Saúde Pública da Lusofonia*’, on 11 and 12 November 2024, specifically targeting the global Lusophone community. This initiative recognizes and celebrates the unique cultural ties among Portuguese-speaking countries, aiming to foster a collaborative environment for public health innovation and discussion.

Also, in anticipation of the conference, we are excited to announce the ‘Sailing to Lisbon 2024’ Webinar series, serving as a prelude to the conference, offering a platform for preliminary discussions, sharing of ideas, and networking among public health professionals. This webinar series is not just a buildup to the conference but an integral part of our journey towards innovation in public health. It offers an opportunity for continuous learning and engagement with the themes that will define the future of public health in Europe and beyond. And there is even an added incentive for participation, as there is a quiz at the end of each webinar, awarding prizes that range from free EPH Conference registrations to entrances for the Conference Dinner and Dance event.

The 17th EPH Conference in Lisbon invites public health professionals and students to navigate the vast ocean of possibilities within our field, charting a course towards a future where innovation and collaboration lead the way in addressing the complex health challenges of our time.

Keep updated on all the details at the conference website: www.ephconference.eu

See you in Lisbon!

